# The Promotion of ‘Grab Bags’ as a Disaster Risk Reduction Strategy

**DOI:** 10.1371/currents.dis.223ac4322834aa0bb0d6824ee424e7f8

**Published:** 2018-07-06

**Authors:** Christina J. Pickering, Tracey L. O'Sullivan, Alessia Morris, Carman Mark, David McQuirk, Emily YY Chan, Emily Guy, Gloria KW Chan, Karen Reddin, Ralph Throp, Shinya Tsuzuki, Tiffany Yeung, Virginia Murray

**Affiliations:** Canada and Overseas Training Fellow, Public Health England, London, United Kingdom; Enhancing Resilience and Capacity for Health (EnRiCH) Research Lab, Interdisciplinary School of Health Sciences, University of Ottawa, Canada; Interdisciplinary School of Health Sciences and Telfer School of Management, University of Ottawa, Ottawa, Ontario, Canada; Scottish Government, Resilience Division, United Kingdom, Edinburgh, Midlothians, Scotland, UK; Collaborating Centre for Oxford University and CUHK for Disaster and Medical Humanitarian Response (CCOUC), JC School of Public Health and Primary Care, The Chinese University of Hong Kong, Hong Kong S.A.R., China.; Emergency Response Department, Health Protection and Medical Directorate, Public Health England, London, United Kingdom; Collaborating Centre for Oxford University and CUHK for Disaster and Medical Humanitarian Response (CCOUC), JC School of Public Health and Primary Care, The Chinese University of Hong Kong, Hong Kong S.A.R., China; Nuffield Department of Medicine, University of Oxford, Oxford, United Kingdom; FXB Centre of Health and Human Rights, Harvard University, Cambridge, Massachusetts, USA; Interdisciplinary School of Health Sciences, University of Ottawa, Ottawa, Ontario, Canada; Collaborating Centre for Oxford University and CUHK for Disaster and Medical Humanitarian Response (CCOUC), JC School of Public Health and Primary Care, The Chinese University of Hong Kong, Hong Kong S.A.R., China.; Emergency Response Department, Health Protection and Medical Directorate, Public Health England, London, United Kingdom; Resilience Division, Scottish Government, Edinburgh, Midlothians, Scotland, United Kingdom; Health Science Division, Minister's Secretariat, Ministry of Health, Labour and Welfare, Japan; Tier 5 Intern, Public Health England, London, United Kingdom; Hong Kong Jockey Club Disaster Preparedness and Response Institute, Hong Kong, China; Overseas Training Fellow for Healthcare Professionals, Public Health England, England, United Kingdom; Public Health England, London, England; Integrated Research on Disaster Risk Scientific Committee, Beijing, China.

## Abstract

Introduction: An all-of-society approach to disaster risk reduction emphasizes inclusion and engagement in preparedness activities. A common recommendation is to promote household preparedness through the preparation of a ‘grab bag’ or ‘disaster kit’, that can be used to shelter-in-place or evacuate. However, there are knowledge gaps related to how this strategy is being used around the world as a disaster risk reduction strategy, and what evidence there is to support recommendations.

Methods: In this paper, we present an exploratory study undertaken to provide insight into how grab bag guidelines are used to promote preparedness in Canada, China, England, Japan, and Scotland, and supplemented by a literature review to understand existing evidence for this strategy.

Results: There are gaps in the literature regarding evidence on grab bag effectiveness. We also found variations in how grab bag guidelines are promoted across the five case studies.

Discussion: While there are clearly common items recommended for household grab bags (such as water and first aid kits), there are gaps in the literature regarding: 1) the evidence base to inform guidelines; 2) uptake of guidelines; and 3) to what extent grab bags reduce demands on essential services and improve disaster resilience.

## 1.0 Introduction

The Sendai Framework for Disaster Risk Reduction 2015-2030 advocates an all-of-society approach toward disaster risk reduction (DRR). This approach recognizes the importance of engagement - across all levels of society - in preparedness activities, including action toward household preparedness^1^.

Household preparedness recommendations are typically oriented toward encouraging people to prepare their households; for self-sufficiency during and after a disaster for at least three days. Grab bags, which can be used to shelter-in-place or evacuate, are commonly recommended as a preparedness strategy^2^. The rationale for recommending assembly of grab bags, is emergency services may not reach everyone within the first 72 hours after an adverse event^3^. A bag of essentials may support self-sufficiency, and ensure the needs of people in a household are met while services are recovering from disruptions. In literature and practice, this type of bag is referred to by different names, including disaster emergency supply kits, disaster preparedness kits, go bags and grab bags. But what is the evidence they work? And how are grab bag recommendations promoted in different parts of the world?

As a first step toward addressing these questions, we present an exploratory study designed to provide insight into how grab bag guidelines are used to promote preparedness in Canada, China, England, Japan, and Scotland, supplemented with a literature review focused on existing research evidence for this DRR strategy. Our objective is to provide an overview of different practices, in combination with existing literature, to inform future research agendas in this area.

## 2.0 Methods

This study is comprised of two parts: the first is a literature review of grab bag guidelines and supporting research evidence and the second is a five-country case study of approaches used to promote grab bags for household preparedness.

For the literature review, peer reviewed publications and grey literature 2006-2016 were searched using two databases (Embase and Scopus), as well as Google. Keywords included: emergency supply kit; emergency kit; go bag; grab bag; disaster supply kit; disaster emergency kit; emergency preparedness kit; disaster; emergency; hazards; evidence; emergency preparedness; resilience; and community resilience. The inclusion criteria were: 1) published in English, 2) focus on household preparedness, and 3) reference to grab bags. We did not include studies focused on general preparedness, or grab bag guidelines specific to pets, businesses and communities. The articles were reviewed and summarized by two co-authors (CP, TO), with emphasis on terminology, evidence of evaluation, and content of grab bags. See Appendix for database search strategies and a summary of search results by database.

The five-country case study included a comparison of approaches used to promote grab bags as a household preparedness strategy. Interns from Canada, China, and Japan, working at Public Health England, conducted research focused on their own countries and England, to contribute to an exchange of preparedness practices. Scotland was included for its novel approach in the establishment of a National Centre for Resilience (NCR).

## 3.0 Results

The results are presented in two parts.

**3.1 Summary of the literature** After removal of duplicates, a total of 38 articles which met the inclusion criteria were included in the literature review. Of these, seven articles were outside the scope of the study, resulting in a final sample of 31 articles. Table 1 summarises the articles included.

Of the 31 articles reviewed, 22 used grab bags as a measure of disaster preparedness. Most of these articles used survey methods to assess population preparedness, using indicators such as having a grab bag and an emergency plan. Facilitators and barriers were commonly discussed, with references to action by different demographics (eg. age, gender, marital status, education).

Bagwell et al.^4^, Crawford and McAlister^5^, Jassempour et al.^6^, Kettunen et al.^7^, Kohn et al.^8^, Kruvand and Bryant^9^, and Mack et al.^10^ evaluated preparedness before and after implementation of community preparedness programs. These studies highlighted techniques and tools with potential to improve awareness and action (eg. preparation of a grab bag or emergency plan). While grab bags were not specifically evaluated, the importance of evaluation for preparedness programs was emphasized.

Five studies investigated medication and medical supplies as important items for a grab bag^11^^,^^12^^,^ 13^,^ 14^,^ 15. The specific needs of high-risk populations are important considerations. Kleinpeter et al.^13^ described the complications that arose for peritoneal dialysis patients after evacuating New Orleans during Hurricane Katrina. Complications regarding storage of stockpiled medication included temperature control and expiration dates. This type of information is important for understanding the specific needs of populations living with chronic conditions, who may be at heightened risk during disasters.

Nine articles did not focus on grab bags as a measure of preparedness, instead they emphasized content and effectiveness of grab bags^16^^,^ 17, disaster preparedness^18^, post-disaster narratives^13^^,^ 19, medical supplies and preparedness^11^^,^ 13^,^ 14, and preparedness interventions^10^^,^^20^. The different research methods, strategies, and approaches to study household preparedness were identified.

Perman et al.^16^ and Heagele^17^ discussed grab bags in terms of the evidence for effectiveness and variations in guidelines offered to the public. Perman et al.^16^ examined the contents of 71 guidelines on recommended items and stated that overly comprehensive lists can be overwhelming, whereas simple lists can be inadequate, calling for a standard for grab bag guidelines including empirical evidence on which items best support resilience. Heagele^17^ reviewed the literature on grab bag guidelines and found a lack of empirical evidence on how they support resilience. Heagele^17^ nevertheless reiterated the importance of continuing to promote grab bags as a preparedness strategy – given their potential to support households needing to be self-sufficient until help arrives. This is particularly important for high risk populations who must tailor the grab bags to ensure they have supplies to meet their unique needs.

**Table 1: Summary of articles:** 

Authors	Purpose of the articles	Focus on grab bags	Terminology
Annis, Jacoby, & DeMers^21^	Evaluate preparedness among US Navy personnel	Grab bags used as a measure of preparedness	Disaster kit; Emergency kit; Emergency preparedness kit; Emergency supplies kit
Goodhue et al.^22^	Evaluate preparedness of families with children in an intestinal rehabilitation clinic	Grab bags used as a measure of preparedness; How many households have a grab bag versus individual items; Extra supplies for children with special needs	Emergency supply kit; Disaster kit; Disaster survival kit
Heagele^17^	Review evidence on effectiveness of grab bags used in household preparedness in the US	Inconsistent reporting; Lack of evidence on effectiveness; Lack of literature on how grab bags items are determined;Facilitators and barriers to preparing grab bags	Disaster preparedness kit; Disaster supply kit
Kruvand & Bryant^9^	Examine whether the CDC* zombie apocalypse campaign translated to preparedness knowledge/ behaviour in youth	Intention to assemble a grab bag used as a measure of preparedness; Strategies to educate youth	Emergency kit
Ochi et al.^14^	Make recommendations on effective preparedness based on a systematic review of medication loss in disasters	Specific items to be included in grab bags: medications and medical aids	Emergency pack; Emergency kit
Tanner & Doberstein^23^	Examine the level of emergency preparedness among University students	Grab bags and individual components used as a measure of preparedness	Emergency preparedness kit; Emergency kit
Thomas et al.^24^	Report on outcomes of CDC employees participating in Ready CDC	Grab bags used as a measure of preparedness	Emergency kit
USAID^19^	Report on how grab bags increased earthquake preparedness in Nepal	Woman’s experience using a grab bag after earthquake was effective	Go bag; Disaster preparedness kit; Emergency kit
Witvorapong, Muttarak & Pothisiri^25^	Examine determinants of and relationships between social participation and disaster preparedness	Grab bags used as a measure of preparedness	Emergency kit
Asada et al.^11^	Survey pharmaceutical patients about preparedness and preservation of medication lists during a disaster	Discusses medication preparation and preservation for diabetes; Suggests keeping a medication lists	Emergency bag
Bagwell et al.^4^	Assess preparedness of families of children with special healthcare needs and the impact of education and interventions	Grab bags used as a measure of preparedness; Participants received backpack with first aid supplies and flashlights	Disaster kit
Chan et al.^26^	Examine if previous disaster experience increases household preparedness in a village in China	Grab bags used as a measure of preparedness	Disaster Emergency kit; Disaster kit
Jassempour et al.^6^	Evaluate the effectiveness of applying a PAPM*-based disaster preparedness education program by focusing on uptake or creation of survival kits	Grab bags used as a measure of preparedness; Measured level of awareness of grab bags and contents	Disaster survival kit
Kohn et al.^8^	Measure outcomes of a personal preparedness curriculum for public health workers	Grab bags used as a measure of preparedness; Linking dissemination with improved uptake; Discussed barriers and facilitators to preparedness behaviours	Emergency kit; Supply kit; Emergency preparedness kit; Preparedness kit
McCormick et al.^27^	Examine the effects of experiencing a tornado on preparedness awareness and personal preparedness	Grab bags and individual grab bag items used as a measure of preparedness pre- and post-tornado	Preparedness kit; Disaster preparedness kit; Emergency preparedness kit; Disaster kit
Gershon et al.^28^	Characterize preparedness for persons with disabilities, determine the role of the personal assistant and the impact of prior emergency experience on preparedness	Grab bags used as a measure of preparedness; Populations changed contents of grab bags after experiencing a disaster	Go-bag; Grab bag
McCormick, Pevear & Xie^29^	Evaluate the level of preparedness of residents and ‘at-risk’ residents, using the mass media personal preparedness ‘Get10’ campaign recommendations	Grab bags and individual grab bag items used as a measure of preparedness	Disaster kit; Preparedness kit; Disaster preparedness kit
Burke, Bethel, & Foreman Britt^30^	Assess knowledge, attitudes, and perceptions about disaster preparedness among Latino migrant and seasonal workers in North Carolina.	Grab bags used as a measure of preparedness	Emergency kit
Loke, Lai & Fung^31^	Explore the extent of disaster preparedness and concerns about disasters among the elderly in Hong Kong	Individual grab bag items used as a measure of preparedness	Survival pack; Emergency survival kit; Supplies kit
Tomio, Sato & Mizumura^15^	Describe disaster preparedness among patients with rheumatoid arthritis and examine how differences in health, functional, and disability conditions are associated with disaster preparedness	Grab bags used as a measure of preparedness; Focus on medications and medical records as a measure of medical preparedness	Emergency pack
Iannucci^20^	Create a prototype person-centered program that individualizes preparedness for persons living in poverty with a disability	Abstract only. Identify and evaluate items needed for a tailored grab bag	Emergency kit; Preparedness kit
Perman et al.^16^	Compare content guidelines for 71 grab bag guidelines in US	Analysis of comprehensiveness and specificity of grab bag guidelines	Disaster kit
Schmidt et al.^32^	Explore perceptions of personal and program preparedness of nursing students	Grab bags used as a measure of preparedness	Disaster kit; Go bag
Semenza, Ploubidis & George^33^	Explore whether the health frame can act as a motivating factor for climate change adaptation behaviour to reduce climate risks	Grab bags used as a measure of climate change adaptation	Emergency kit
Crawford & McAlister^5^	Prepare a high-risk population for disaster	Grab bags used as a measure of preparedness; Distributed 3000 grab bags	Go-bag
Kettunen et al.^7^	Evaluate educational efforts of a pandemic preparedness committee and assess community readiness	Grab bags used as a measure of pandemic preparedness	Emergency kit
Feret & Bratberg^12^	Assess views of preparation and readiness of assisted-living residents after participating in a preparedness program	Grab bags used as a measure of preparedness; Focus on medical information and supplies	Disaster kit; Emergency preparedness kit; Emergency kit
Department of Homeland Security^18^	Update and summarize current citizen preparedness research	Comparison of grab bag surveys from 2005-2007; Review of studies using grab bags as a measure of preparedness	Disaster supply kit; Emergency preparedness kit; Go bag; Disaster kit; Emergency supply kit
Kleinpeter, Norman & Krane^13^	Describe disaster planning, implementation, and follow-up that occurred in a PD* program after Hurricane Katrina	Patients told to bring 1 week of PD supplies. No other grab bag contents specified	Disaster kit
Mack et al.^10^	Introduce a curriculum that prepares low-income, low-resource families to survive disaster	Discusses barriers to grab bag assembly; Emphasizes need for items for children and culturally diverse food lists	Disaster kit; Safety kit; Disaster preparedness kit; Preparedness kit; Disaster survival kit
McRandle^34^	Provide tips and environmentally sensitive solutions to help people manage in a disaster	Discusses items to include and the need to check items regularly	Emergency kit

*Abbreviations: CDC= Centre for Disease Control; PAPM= Precaution Adoption Process Model; PD= Peritoneal dialysis

****3.2 Summary of the five-country case studies ****The five country case studies, explored grab bag guidelines in Canada, China, England, Japan, and Scotland. The institution of origin for grab bag guidelines and recommendations varies across each country. In Canada, Japan, and Scotland, grab bag guidelines and recommendations stem from national government through Public Safety Canada, the Fire and Disaster Management Agency (FDMA), and Ready Scotland, respectively. In China, grab bag guidelines and recommendations occur through an academic institution from the Collaborating Centre for Oxford University and CUHK for Disaster & Medical Humanitarian Response (CCOUC). In England, grab bag guidelines and recommendations are the responsibility of individual local authorities through Local Resilience Forums (LRFs) such as the Coventry, Solihull and Warwickshire (CSW) Resilience Team. These sources and their grab bag guidelines were explored in each country.

**3.2.1 Canada** In Canada, disaster preparedness is coordinated at a national level by Public Safety Canada^35^. Their website includes guidelines on what to include and the use of grab bags. This is an integral part of an ongoing campaign called ‘*72 Hours… Is Your Family Prepared?*’ that encourages households to be prepared to be self-sufficient for at least 72 hours after an adverse event^3^. In addition to grab bag guidelines, the campaign includes tips on knowing the risks in your community, and making a household emergency plan. A video explains how to assemble household grab bags and includes an interactive checklist. The 72 Hour campaign also provides a specialised grab bag guideline for persons with disabilities, who may have specific needs in an adverse event.

Public Safety Canada designates grab bags as emergency kits. The following is a list of items suggested: water, food, a manual can opener, wind-up battery-powered flashlights, a radio with extra batteries, first aid kit, extra keys for the car and house, cash, travelers’ cheques and change, and important family documents (e.g. identification, insurance, bank records, a copy of the household emergency plan, and contact information)^36^. Additional supplies to consider are: water for cooking and cleaning, personal hygiene items, hand sanitizer, toilet paper, prepaid phone card, mobile phone charger, pet food, infant formula, baby food and supplies, prescription medications, medical equipment, a whistle, and duct tape^36^. Within both lists, there are justifications for items included and website links are included for organizations that have grab bags available for purchase (e.g. St. John Ambulance, Salvation Army, and the Canadian Red Cross).

In addition to publications, the 72 Hour Preparedness campaign uses a range of dissemination techniques through promotional materials, social media, advertising, exhibits and special events, such as the annual Emergency Preparedness Week in May^3^. According to the Government of Canada^3^, the *Get Prepared* website has been visited 3 million times since its launch in 2006.

Other organizations in Canada that provide online guidelines for preparing grab bags are the Canadian Red Cross, Scouts Canada, Girl Guides Canada, and individual provincial/territorial governments and municipalities. Of note, the quantity and types of items suggested varies by organization, with some commonalities, such as water and food. There are extensive guidelines from organizations within Canada describing items to include in household grab bags and how they might be used, however there are inconsistencies, particularly in terminology. For example, the Canadian Red Cross calls them disaster preparedness kits^37^, while Emergency Management Ontario refers to them as emergency survival kits^38^.

While all organizations utilize their websites to disseminate disaster preparedness information, some are more proactive at communicating to the public. The Canadian Red Cross (@redcrosscanada) and Public Safety Canada (@Get_Prepared) for example, use Twitter to promote preparedness. Additionally, The Canadian Red Cross created a preparedness app called ‘Be Ready’ which aims to spread awareness of the importance of preparedness while giving people a tool to help prepare for and act in a disaster^39^.

**3.2.2 China** The CCOUC was established by the joint effort of Oxford University and the Chinese University of Hong Kong as a non-profit research centre. One of its core missions is to provide knowledge transfer and community service to enhance preparedness among communities facing disaster threats in Greater-China and the Asia-Pacific region^40^. Their Ethnic Minority Health Project (EMHP) is an initiative that highlights the work of CCOUC in disaster preparedness^41^. In China, there are no governmental level guidelines on using grab bags, therefore CCOUC works to fill this gap by providing information about household grab bags, with support from local health authorities, such as the Chinese Center for Disease Control and Prevention, and village heads and village doctors. CCOUC advocates for the preparation and use of grab bags to minimize negative health impacts of natural disasters, whilst EMHP targets ethnic minorities residing in remote, poor, and disaster-prone areas.

Recommendations for grab bags, prepared by CCOUC in 2009, are based on the main protection principles highlighted by the Sphere Handbook^42^. The recommendations address basic survival needs for health, and the unique environmental constraints of rural areas in China. These include: a) water, sanitation and hygiene, b) food and nutrition, c) shelter and non-food items, d) healthcare and e) access to information. All items contained in the grab bag can be adapted to various hazard and disaster contexts. For example, the multi-purpose knife serves various functions (e.g. can opener, cutter, direction indicators) and the flint, apart from lighting a fire, can send signals allowing relief workers to locate victims^43^.

CCOUC emphasizes that decisions regarding what to include in a grab bag be based on public health principles and individual needs. For example, it is important to consider the special needs of patients living with chronic conditions^44^ and/or those who have lower literacy skills. CCOUC asks patients to take pictures of the medications they are currently taking; this helps ensure relief workers can efficiently and accurately identify medications people are taking and support patient safety. Regarding information and communication, CCOUC encourages villagers to keep a family portrait, a list of emergency contact information, and a copy of all the identification documents for each of the family members as part of their household grab bag. The family portrait and emergency contact information are particularly useful to support communication among people with low literacy skills. Having a copy of identification documents is useful if evacuation is required, health services are needed, and original documents are inaccessible^43^. To ensure villagers have a clear understanding of the importance and use of grab bags, CCOUC distributes grab bags in the EMHP sites and provides education on how to prepare, adapt, make accessible and use them according to local context^43^. Figure 1 provides an example of the grab bag and grab bag contents distributed to rural villages in China by CCOUC.

**Grab bag and contents distributed to rural villages in China by CCOUC:**
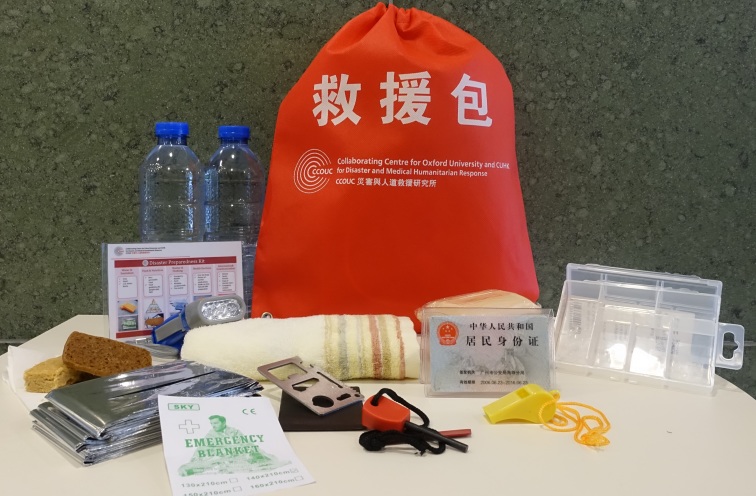
Picture taken by co-author (GKWC) and may be published under the CC-BY license with permission from the copyright holder (GKWC), original copyright 2016.

**3.2.3 England** In England (and the United Kingdom) emergency preparedness is primarily governed by the 2004 Civil Contingencies Act (CCA). This places duty on several agencies to prepare for and assist the public in preparing for emergencies, such as widespread flooding. The first publication to include a reference to an emergency grab bag was the leaflet Protect and Survive^45^.

In 2004, the Government produced and sent a leaflet to all residents^46^ that detailed actions to be taken in the event of an emergency, and included a list of suggested contents for a household grab bag. These suggested contents included: a list of useful phone numbers; home and car keys; toiletries, sanitary supplies and regularly prescribed medication; a battery radio and a torch with spare batteries; candles and matches; a First Aid kit; a mobile phone; cash and credit cards; spare clothes and blankets; bottled water, ready-to-eat food and a bottle/tin opener. This list has remained effectively the same for the past decade, but with added emphasis on tailoring the contents for individual needs (e.g. specific supports for children, older people, persons living with disabilities and pets).

The use of grab bags is now currently promoted locally. For example, an LRF, based in Bedfordshire (BLRF), uses Twitter (@what_would)^47^ to engage with the community and re-tweets examples of grab bag good practices from around the country including Merseyside^48^ and Essex^49^. Another LRF in Coventry, Solihull and Warwickshire (CSW) use their website^50^ and Twitter account (@PreparedPics) to promote the use of grab bags with a series of tweets throughout June 2016^51^ using the #GrabBag hashtag and went through recommended items for a grab bag in an informative, but light-hearted manner.

The way guidelines for grab bags are presented varies considerably across England. CSW uses a light-hearted manner across their website and Twitter presence with a family of cartoon characters based on crash test dummies to emphasise the points being made^52^. Essex has communicated the importance and use of grab bags through their “What If” initiative^53^. Local Resilience Fora use branding for all preparedness information and warnings, are actively promoting the use of grab bags, and strategically aligning all messaging for consistency and recognition. There are 38 LRFs in England. Each local fora has grab bag guidance, such as Bedfordshire^54^, however Essex and CSW are more prominent.

In London, a grab bag display can be found in the Natural History Museum, in their Volcanoes and Earthquakes Exhibition (see Figure 2). Using ‘go-bag’ as their terminology of choice, the simplistic and comprehensive display summarises the goal of grab bags: to provide food, water, shelter, warmth, communication, tools, first aid, and hygiene. The display includes physical examples of the items, such as a wind-up radio, waterproof notebook and the grab bag itself. The grab bag is defined in relation to people living in earthquake zones and can be found in the exhibit about the deadly 2011 earthquake and tsunami in Japan.

**Go-bag display in the Volcanoes and Earthquakes Exhibition at the Natural History Museum in London, England.**
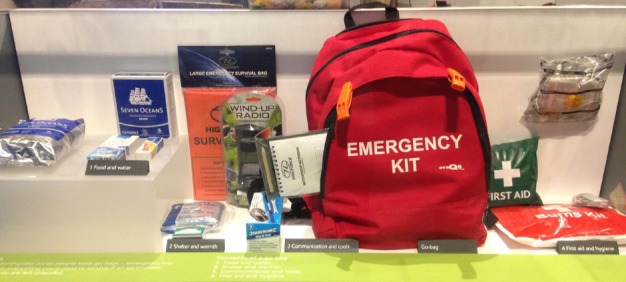
Photo taken by the lead author (CJP) with permission from the Natural History Museum. Image may be published under the CC-BY license with permission from the copyright holder (CJP), original copyright 2018.

**3.2.4 Japan** In Japan, the FDMA, a subordinate body of the Ministry of Internal Affairs and Communications, oversees community education and interventions to promote disaster resilience. One DRR strategy used by this organization is to provide grab bag guidelines^55^.

The grab bag contents suggested by the FDMA^55^ include: a bankbook, flashlight, candles, gloves, radio, dry battery, cash, cigarette lighter, can opener, helmet, disaster hood (used as a cushion daily, or used as a hood), knife, baby bottle, food, blanket, clothes, first aid kit, and their signature stamp. It is common for the Japanese to use a signature stamp in formal documentation procedures. FDMA also provides a disaster survival handbook on their website as educational material providing detailed recommendations about grab bag contents. For example, diapers and sweaters as suggested clothes, and food, such as instant noodles, chocolates, and canned food^56^.

The FDMA handbook has various advantages. Firstly, it can be accessed electronically. Secondly, cartoon images are used in each chapter to make it easier to understand for people with limited literacy skills or for whom Japanese is not their first language. Only a limited portion of the official website is translated into English, therefore it would be difficult for people who cannot read Japanese to access the webpage.

**3.2.5 Scotland** Scotland is pursuing a multi-dimensional, ‘whole society’ approach to building resilient communities, which aims to make resilience everyone’s business, summarized in ‘Ready Scotland’^57^. This brings together a wide range of organisations in the public, private, voluntary sector organisations and other civil society groups in a collective effort to change the culture around resilience, and improve the ability of communities to prepare for, respond to, and recover from emergencies.

The Scottish approach is delivered through an assets-based community development approach by Scotland’s resilience community. This means providing individuals and groups of people with the knowledge and skills they need to effect change in their own communities, through a process of engagement, education, empowerment and encouragement^58^. The focus is on relatively simple ‘asks’ of individuals, organisations and communities, with the intention that they a) become more aware of risks, b) plan and take action toward preparedness, and c) cooperate with and help others. The public are encouraged to assemble grab bags to improve household resilience. In empowering self-sufficient citizens, this strategy allows the public to contribute to the resilience of their communities, allowing emergency response agencies to more accurately prioritize cases such as addressing the needs of high risk populations. The “Ready Scotland” website hosts advice on grab bag contents, and household emergency plan templates which include a checklist of items^59^. This advice has been taken up, adapted and used by a range of partner organisations.

The National Centre for Resilience (NCR) acts as a “hub” for the Scottish resilience community providing research, analysis and leadership in developing best practices. It facilitates shared outcomes and priorities in community resilience by supporting partners through design, delivery and dissemination of resources and toolkits such as grab bags^60^. The NCR is a catalyst for collaborations, by bringing together resilience partners on a network basis across diverse communities, including those in educational settings. Resilience education initiatives, in formal and informal settings, have adapted the grab bag concept to be more youth-friendly. For example an activity based around completing a “Family Emergency Plan”, which includes a grab bag checklist to be completed by families or in the classroom.

## 4.0 Discussion

This paper presents a literature review focused on the use of grab bags to promote household preparedness and five country case studies of grab bag promotion. Though the academic literature has identified a gap in empirical evidence for this DRR strategy, promotion of grab bags continues. Indeed the authors who contributed to featured case studies support grab bag strategies, recognizing the approach’s potential to save lives and reduce negative disaster impacts. This strategy reflects the precautionary principle; which stipulates that risk management action can be taken despite scientific uncertainty due to lack of evidence, to protect people from harm^61^. Based on this principle, we recommend that grab bags continue to be promoted as a household preparedness measure, but echo recommendations by Perman et al.^16^ and Heagele^17^ for an improved evidence base and development of good practice alongside its current use.

**4.1 Evidence of grab bag effectiveness** The Sendai Framework stresses engagement with its recommendations for an all-of-society approach to DRR^1^. The assembly of grab bags, tailored to the needs of individuals in a household, exemplifies activity that promotes discussion of preparedness and has the potential to move people toward action. Grab bags as a preparedness measure are promoted by many countries to reduce the risk of harm to a population in the face of a hazard and are assumed to influence resilience, but further evidence is needed to understand this role.

Two of the 31 articles reviewed explicitly provided information on the effectiveness of grabs bags for increasing community resilience. Heagele^17^ discusses the lack of evidence on the efficacy of grab bags and the need for further research to support assumptions of grab bags contribution to DRR. The author stipulates that it is important to investigate if and *how* grab bags are effective. A recent article from USAID^19^ includes testimony from a woman who used a grab bag during the deadly Nepal earthquake in April 2015 who discusses how the items helped her family survive post-disaster.

Research examining the effectiveness of grab bags as a preparedness behaviour would be beneficial to: 1) inform policy makers, government officials, and investors about the potential return of investment on promotional grab bag campaigns; 2) enhance motivation for grab bag preparation; and 3) improve knowledge about the most useful items to include in a grab bag. A research agenda should include qualitative methods to capture experiences of peoples’ use of grab bags. These methods could improve understanding of grab bags usage and how to move people from awareness to action; grab bags might also influence public confidence regarding whether preparedness actions will make a difference in a disaster^18^. An additional item for the research agenda is to explore mechanisms that contribute to uptake of grab bag recommendations, while also exploring different strategies to include households with limited means to assemble these types of kits.

**4.2 Content of grab bags** When using grab bags as a measure of preparedness, survey questions most often ask about having a grab bag, rather than individual items. However, Goodhue et al.^22^, Loke et al.^31^, McCormick et al.^27^^,^^29^, and Tanner and Doberstein^23^ inquired about specific types of supplies in the grab bag. This distinction provided insight not only about who possessed a grab bag, but also its comprehensiveness, and which items were easiest to acquire.

This literature review revealed that grab bags are an integral DRR strategy, and often used as an indicator for preparedness. The literature is predominantly quantitative, and focused on facilitators and barriers to preparedness at household and individual levels. The quantitative studies that examined grab bag use, by investigating completeness of grab bags compared to official guidelines, provide a base for future research. Qualitative and mixed methods studies are needed to better understand the role of grab bags in supporting community resilience.

**4.2.1 Listing content for guidance **In the case studies, guidelines varied according to the type of items and quantity. Some guidelines focused on portability, for utility in evacuation, whereas others included too many items to be realistically portable. This issue was identified by Perman et al.^16^ where some of the 71 checklists they compared discouraged action because they were overwhelming. Given that grab bags are promoted for both sheltering-in-place and evacuation, the need for realistic lists and evidence to support them is paramount.

**4.2.2 Approaches to communication **Another area of variability, noted in the five-country case study, is the accessibility of the grab bag guidelines and dissemination techniques. While all five countries utilize organizational websites to display grab bag guidelines, dissemination techniques vary. In Canada, grab bag promotion is incorporated into a larger preparedness campaign titled ‘*72 Hours… Is Your Family Prepared?*’ using a variety of strategies such as promotional material, social media, and special events^3^. In China, the CCOUC uses websites and leaflets to disseminate information on grab bags, while also using knowledge transfer initiatives such as the EMHP. Through EMHP, CCOUC distributes physical grab bags to some high risk populations. In Scotland, dissemination strategies include the ‘Ready Scotland’ campaign, flyers, and preparedness activities in schools. England and Japan use similar dissemination strategies using cartoons to add a fun and light-hearted tone to their message. In England, the CSW Resilience Team regularly uses Twitter to promote grab bag use using cartoon families. In Japan, the FDMA government website is home to information on grab bags and an electronic disaster survival handbook that uses cartoons and drawings of grab bag items to make the handbook more accessible for persons with lower literacy, or those who cannot read Japanese.

The five-country case study revealed promising practices to promote awareness and action. Empirical studies to understand the uptake of recommendations would enable organizations to invest in practices that have higher impact on behavioural outcomes. The literature has repeatedly shown that a small percentage of the population is prepared and has a formal grab bag ready^18^^,^^22^^,^^31^^,^^32^. Yet of those who have grab bags, many do not contain all the recommended items^22^^,^ 23 ^,^ 27 ^,^ 29. This underscores the need to evaluate dissemination and public interpretation of preparedness guidelines. A number of studies have started to address this gap, with evaluation of tools, programs and dissemination techniques for changes in knowledge and uptake of preparedness behaviours such as grab bag preparedness^6^^,^ 7 ^,^ 9 ^,^ 10.

**4.3 Limitations** This study provides insight from five-country case studies and a review of extant literature. While grab bag guidelines were explored across countries, the variation in terminology, contexts, and disaster risk sensitivities makes direct comparison challenging. The differences in contexts may explain inconsistencies in suggested items for inclusion in a grab bag. Expansion of this study to other countries, particularly developing countries, is recommended. Additionally, due to resource constraints, the literature review only searched two bibliographic databases and Google. A next step would be to expand the literature search to include other databases and languages.

## 5.0 Recommendations

The following recommendations are provided to address gaps in the literature on effectiveness of grab bags, and share promising practices in household preparedness strategies. This study supports the continued use, promotion, and preparation of grab bags, despite lack of evidence of effectiveness. To build an evidence-base, more research is needed to understand: 1) the evidence base that informs guidelines; 2) uptake of grab bag guidelines at the household level; and 3) to what extent grab bags can reduce demands on essential services and improve disaster resilience. Such research can provide insight on how to encourage public engagement in disaster preparedness practices and support community resilience processes.

Future research should also continue to examine dissemination techniques to ensure guidelines are reaching intended audiences and promoting positive change in preparedness behaviour. Tailored messaging will ensure the needs of different populations are included, particularly for households where there are limited means to assemble supplies.

Defined terminology is an important aspect for grab bag guidelines, the most common terms used were ‘disaster kit’, ‘emergency kit’, ‘disaster preparedness kit’, and ‘emergency preparedness kit’. Introducing standardized terms could help to minimize confusion and provide consistency for sharing information between countries and organizations. An additional recommendation is to add the term ‘grab bag’ to the glossary of terms provided by leading organisations in DRR such as the World Health Organization (WHO).

## 6.0 Conclusion

The results of this study identified gaps in the evidence on the effectiveness of grab bags, and found variations in guidelines and promotion practices across different countries. With the implementation of the Sendai Framework and its emphasis on an all-of-society approach to DRR^1^, there is an opportunity to raise widespread awareness of the importance of household preparedness. Grab bags are recognized as an important strategy to support DRR, however the need for an evidence base must be addressed to support investments in this area.

## 7.0 Appendix

**Table 2: Embase search strategy:** 

#	Searches	Results
1	("grab bag*" or grab-bag* or "go bag*" or GoBag* or go-bag* or "bug out bag*").tw.	19
2	((disaster or emergenc*) adj2 (kit* or bag* or pack*)).tw.	385
3	1 or 2	404
4	((surviv* or evacuat* or disaster* or emergenc* or hazard*) adj3 (plan* or prepar*)).tw.	9931
5	disaster/ or disaster planning/	21453
6	4 or 5	28499
7	3 and 6	46
8	limit 7 to ((chinese or english or japanese) and yr="2006 -Current")	39
9	(us* or stor* or cont* or supplies or composition or inclu* or prepar* or list* or checklist* or guideline*).tw.	12274294
10	8 and 9	37
11	(effective* or resilien* or efficien* or benefi* or impact or success* or useful*).tw.	4501740
12	8 and 11	19

## 

**Scopus search strategy:**
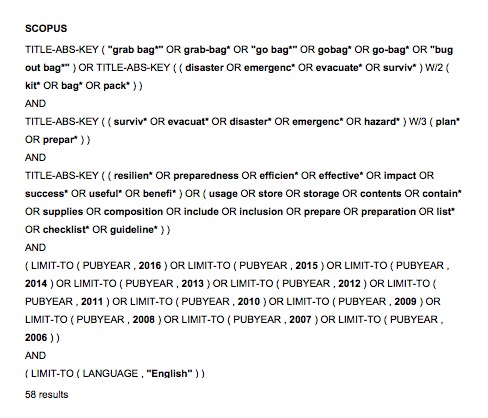
Screenshot taken by the lead author (CJP). Image may be published under the CC-BY license with permission from the copyright holder (CJP), original copyright 2018.

## 

**Table 3: Summary of databases searched and results:** 

Source	Results
EMBASE	39 (27 relevant)
SCOPUS	58 (22 relevant)
TOTAL (after removing duplicates)	35

## Corresponding Authors

Christina J. Pickering (cpick030@uottawa.ca) and Dr. Virginia Murray (Virginia.Murray@phe.gov.uk.)

## Data Availability Statement

All relevant data are within the manuscript.

## Competing Interests Statement

I have read the journal’s policy and have the following conflicts to declare. The following co-authors of this manuscript are currently members of the Editorial Board for a PLOS Currents journal: Dr. Emily YY Chan, Dr. Tracey O'Sullivan, and Dr. Virginia Murray. The authors have no other competing interests to declare.
